# Forced migrants involved in setting the agenda and designing research to reduce impacts of complex emergencies: combining *Swarm* with patient and public involvement

**DOI:** 10.1186/s40900-017-0073-z

**Published:** 2017-11-06

**Authors:** Julii Suzanne Brainard, Enana Al Assaf, Judith Omasete, Steve Leach, Charlotte C. Hammer, Paul R. Hunter

**Affiliations:** 1 Norwich Medical School, University of East Anglia, Norwich, UK; 2 School of Pharmacy, University of East Anglia, Norwich, UK; 3 School of Development Studies, University of East Anglia, Norwich, UK; 4 Emergency Response Department, Public Health England, Porton Down, Salisbury, UK

**Keywords:** Forced migrants, Refugees, Complex emergencies, Agile development, Mental health, PPI representatives

## Abstract

**Plain English summary:**

The UK’s National Institute for Health Research (NIHR) Health Protection Research Unit in Emergency Preparedness and Response was asked to undertake research on how to reduce the impact of complex national/international emergencies on public health. How to focus the research and decide on priority topics was challenging, given the nature of complex events. Using a type of structured brain-storming, the researchers identified the ongoing UK, European and international migration crisis as both complex and worthy of deeper research. To further focus the research, two representatives of forced migrant communities were invited to join the project team as patient and public (PPI) representatives. They attended regular project meetings, insightfully contributed to and advised on practical aspects of potential research areas. The representatives identified cultural obstacles and community needs and helped choose the final research study design, which was to interview forced migrants about their strategies to build emotional resilience and prevent mental illness. The representatives also helped design recruitment documents, and undertake recruitment and interviewer training.

**Abstract:**

**Background:**

Many events with wide-ranging negative health impacts are notable for complexity: lack of predictability, non-linear feedback mechanisms and unexpected consequences. A multi-disciplinary research team was tasked with reducing the public health impacts from complex events, but without a pre-specified topic area or research design. This report describes using patient and public involvement within an adaptable but structured development process to set research objectives and aspects of implementation.

**Methods:**

An agile adaptive development approach, sometimes described as *swarm*, was used to identify possible research areas. Swarm is meant to quickly identify strengths and weaknesses of any candidate project, to accelerate early failure before resources are invested. When aspects of the European migration crisis were identified as a potential priority topic area, two representatives of forced migrant communities were recruited to explore possible research ideas. These representatives helped set the specific research objectives and advised on aspects of implementation, still within the swarm framework for project development.

**Results:**

Over ten months, many research ideas were considered by the collaborative working group in a series of six group meetings, supplemented by email contact in between. Up to four possible research ideas were scrutinised at any one meeting, with a focus on identifying practical or desirable aspects of each proposed project. Interest settled on a study to solicit original data about successful strategies that forced migrants use to adapt to life in the UK, with an emphasis on successfully promoting resilience and minimizing emotional distress. “Success in resettlement” was identified to be a more novel theme than “barriers to adaption” research. A success approach encourages participation when individuals may find discussion of mental illness stigmatising. The patient representatives helped with design of patient-facing and interview training materials, interviewer training (mock interviews), and aspects of the recruitment.

**Conclusion:**

Using patient and public involvement (PPI) within an early failure development approach that itself arises from theory on complex adaptive systems, we successfully implemented a dynamic development process to determine research topic and study design. The PPI representatives were closely involved in setting research objectives and aspects of implementation.

## Background

Health care and health care delivery are acknowledged to be increasingly complex [1], with outcomes that are often unpredictable and can even be paradoxical. Inconsistent inputs, ever-changing active agents and institutions, unforeseen relationships and consequences are common aspects of real life public health problems. In response to these challenges, many articles have advocated using the lens of complexity science to improve health care delivery [1–8]. Many definitions of complex systems exist, which all identify the properties of a system that has many interacting parts that lead to global behaviour that cannot easily be explained in terms of understanding interactions between the individual constituent elements.

Recognizing the inherent properties of complex systems and complex health problems, may suggest how to design more effective health care delivery [1, 2, 7]. The merits of a perspective informed by complexity science (CS) in public health appears to be strongly supported by pragmatic acceptance of the nature of real life situations, and also a useful counter-balance to the potential weaknesses in reductionist perspectives, and potentially over-optimistic reliance on supposed evidence-based medicine [1–3].

The UK National Institute for Health Research (NIHR) funded the Health Protection Research Unit in Emergency Preparedness and Response from 2014 to 2019, with (among other objectives) a remit to improve the evidence base for reducing the impact on public health from complex events. The NIHR requires its grant holders to provide a voice for populations targeted by NIHR research, in the form of public and patient involvement (PPI) representatives [9]. The inputs of PPI representatives can be diverse [10], but are most often limited to advising (during a single consultation session) researchers on aspects of implementing studies, where research objectives have already been set – at least broadly. This article reports how our group went one step back and collaborated with PPI representatives over multiple meetings in order to set priorities, determine study objectives, revise aspects of implementation and participate in researcher training, using a dynamic project design process.

*Swarm* or a “swarmware” development approach, has been recommended to address complex problems, or problems within complex adaptive systems (such as health service provision) [11, 12]. In practice, Swarm can mean a repeated cycle of collaborative and open-minded intuitive proposals for further development, tested by rapid searches for background information, rapid testing of proposed methods and input data, with critical review to assess feasibility or weaknesses in the proposed work plan. Swarm as a development process is well-established in IT product development, and is closely allied with other IT development philosophies such as sprint [13] and agile development [14], either of which are often preferable to the clockware [12], waterfall or linear sequential approaches to project design [15]. The premise of linear sequential planning is that the development process and objectives are determined before work begins; therefore whether or not the results will be satisfactory may not be apparent until late in the project timeline, after resources have been heavily invested. Linear sequential planning can be appropriate in situations where requirements are well understood, but may stifle innovation, especially in response to rapidly changing circumstances, and thus fail spectacularly and suddenly due to unforeseen conditions. Swarm as a process to be used in designing health-related research or delivering health care was endorsed by Richard Smith [16], previous editor of the *British Medical Journal*. Table 1 contrasts some of the most salient differences between swarm and a clockware approach, especially in the context of research design. Clockware and swarm are *not* mutually exclusive development philosophies; most real life projects use elements of both (we also used elements of both). However, the steady planned deep analysis strategy characteristic of clockware is much more familiar to most health researchers. Note that this table is just one of many possible interpretations of the differences, and is by no means comprehensive.

**Table 1:** Characteristics of clockware and swarm development strategies

Clockware	Swarm
Planned by following protocols	Dynamic planning, in response to uncertain or changing environment
Linear planning	Rarely linear, usually non-linear planning
Detailed and careful planning before start	Early planning is rapid with just enough ideas to allow early testing
Ideas developed in detail before testing and then small modifications if problems arise	Ideas tested early and discarded if not suitable; new ideas generated and tested. Process repeats until ideas sustain repeat testing and refinement without failure
Resources are distributed to tackle different parts of the problem separately	Resources are concentrated together on each stage or part of the problem (like a swarm of bees)
Standard operating procedures and checklists used to generate ideas and specify methods	Brainstorming what to do and how to do it
Fixed time table	Open time table
Deep analysis	Rapid analysis
Early failure is seen as obstacle	Early failure is an opportunity
Assumes static, closed system	Assumes open, unpredictable system
Problem to solve is viewed as finite	Problem is viewed as infinite
Overall effort to control process	Overall effort to find best responses
Process leads to consistent outcomes	The process changes itself and the outcomes, so outcomes will be inconsistent
Failure may not be apparent until implementation	Failure is sought at every stage
Feedback and testing focused on pilot testing and final evaluation	Feedback and testing at all stages

## Methods

This work was undertaken in the city of Norwich, UK. A swarm process was adopted by the investigators to develop research plans. At regular group meetings, up to three research ideas were elicited from the research team (comprised initially of nine individuals). Between meetings, rapid literature searches were undertaken to look for similar or related previous research, as well as determine if relevant research gaps existed. Rudimentary research plans were assembled (by JSB) from these searches and circulated among team members, with feedback elicited by email. The group comments were discussed at the next meeting and further revisions requested to existing ideas or new research ideas elicited. When interest developed in health impacts linked to a specific target group, steps were taken to recruit suitable PPI representatives on (initially) a temporary basis. The PPI representatives were incorporated into the brainstorming and rapid testing process, invited to give feedback at both meetings and via email contact between meetings. Early meetings with the PPI representatives were exploratory, to test the feasibility of potential research directions involving their input. As the feasibility of a specific study became clearer, the PPI representatives progressed to roles of helping to define the research objectives, design recruitment materials, comment on aspects of implementation and participate in researcher training.

## Results

Some example research ideas considered but ultimately discarded by the team using Swarm are listed in Table 2. There was an overlapping period when some of these ideas were still under development even as we also considered a research plan addressing complex health needs of forced migrants (especially people involved in the ongoing European Migrant Crisis [17–19]). However, the team was acutely aware that we lacked personal experience of this target group or understanding of their perspectives. It was desirable to recruit PPI representatives who could both bring perspective and advise if any of our subsequent research ideas were good ones, or indeed how we could create a culturally sensitive research plan.

**Table 2:** Early research ideas not pursued by the working group

Research question and possible study design	Group comments on relevant challenges, issues of concern upon implementation
Modelling to test UK Health system capacity, focus on demand surges	The people most affected are those already most vulnerable; learning how to reduce the general ill health burden among such groups could be more useful.
Modelling to test UK Health system capacity, focus on combined inputs (surges and conditions)	As previous; specific scenario development needs to involve multiple partners; extensive literature search required to identify knowledge gaps. Local Resilience Partnerships (groups of organisations tasked with promoting recovery after a severe event) could be consulted, but their network development is regionally inconsistent.
Modelling optimally early detection when events are beginning to cascade towards a severe incident	Describes existing role of syndromic surveillance systems; had undesirable potential to overlap too much with remit of colleagues' (other Health Protection Research Unit) groups.
Modelling of input of resurgence of existing contagion or new infectious disease	Would need to incorporate efficacy of existing syndromic surveillance systems (not well understood, requires own research).
Modelling of burden of unnecessary presentations to health care professionals after public health scares	Inappropriate presentation is often difficult to define. Also inappropriate presentation is a difficult problem to modify, for instance when official advice is to seek medical advice, given to patients with symptoms matching a genuine public health concern.
Historical review of past very large events to identify range of input stressors	Would be useful to one of the above models; long term consideration for further research.

Several local organisations with responsibilities for supporting refugees and asylum seekers were asked (by JSB) to help us recruit PPI representatives. Only the British Red Cross (Norwich office) was responsive to our queries. The Red Cross nominated three individuals they were in contact with, two of whom were available to participate as our PPI representatives. These PPI representatives each had roots in parts of the world supplying the majority of the forced migrants in the European migration crisis: the Middle East (Syria) and sub-Saharan Africa (Kenya). Author JO was an international development student who also worked for the local branch of the British Red Cross, supporting forced migrants. Author EA was a service user rather than an employee of the Red Cross, and had a background in pharmacology, having trained as a hospital pharmacist in Syria. She subsequently became a PhD student in pharmacology. These academic and professional backgrounds helped the PPI representatives to bridge the gap between our investigator perspectives and those of their cultural communities. Both PPI representatives were female (unlike most of the investigators), fluent English speakers, which was essential for collaboration, as well as had assured right to remain in the UK (valuable for them to have a medium-long term relationship with our research group). Author JSB handled related institutional paperwork and acted as the main liason between the PPI advisors and the research team.

The PPI representatives were invited initially as temporary advisors, but with the potential for longer term input if they saw utility in any proposed research directions relevant to their representative roles. Meetings were held at a location and at times and on days that were mutually convenient for the key researchers (authors JSB, PRH and SL) and both representatives (authors EA and JO). The advisors were each paid a set fee (about three times the minimum wage) for each meeting they attended (typically one hour long). This reimbursement also covered their involvement between meetings (answering emails and reading material). Drawing on expertise within the group and from rapid literature scans, we knew there were research gaps for immigrants in improving sexual health, reducing infectious disease transmission and addressing mental illness. The PPI representatives advised on social stigma perceived by forced migrants about all of these potential health problems. The collaborative group saw an opportunity to research these areas using positive reframing. Positive reframing means, as much as possible, addressing a sensitive and potentially problematic issue using positive and non-stigmatic messages and presentation [20, 21]. Following discussions of cultural and practical data collection obstacles, the group felt that mental illness had the most potential for original data collection using positive reframing.

In the EU and the UK, the majority of asylum applicants tend to be physically healthy young adults without chronic physical diseases. However, these asylum seekers and recipients have specific and complex vulnerabilities. Post traumatic stress disorder and other mental health conditions directly linked to civil conflict and displacement are widely recognised among this group [22–25]. Refugees and asylum seekers have both complex health care needs and the disadvantage of having to learn new ways of how to access health care in an unfamiliar (and complex) health care system. Difficult experiences post-trauma, including during and after resettlement in a ‘safe’ country, are understood to strongly increase the risks of a forced migrant developing mental illness [26–30], perhaps because this is a group at high risk for reduced resilience.

Colleagues with expertise in qualitative interview methods (including author CCH) joined our research planning team to further develop the research plan, including to advise on positive reframing. Rapid literature scans again suggested that most research on mental illness experienced by forced migrants is mostly observational and quantitative (eg., how many people are affected [25, 27, 31]) or usually focuses on barriers to accessing mental health services [32–34], and thus uses negative framing. Some research detailing protective factors for emotional resilience exists [28, 35, 36], but few studies have collected original data about emotional resilience among forced migrant adults in the UK.

### Aspects of study design and implementation

Together the working group designed a study to collect original information about emotional resilience among forced migrants, using individual face to face interviews. The PPI representatives advised against a group discussion format in case it inhibited candid discussion (in front of fellow migrants who might also be friends and family). Relatively private interviews also meant that it would be easier for the researchers to maintain positive framing about potentially stressful emotional experiences that participants may have had. The PPI advisors and the investigators agreed that the research should recruit participants who were not asylum seekers but rather already had assured status (asylum granted) in the UK. People without guaranteed status might be reluctant to participate due to fear of possible impacts on their asylum application. Moreover, those who have been in the UK for a longer period were more likely to be able to contribute to positive reframing rather than become re-traumatised when asked to reminisce.

The PPI representatives preferred the term “forced migrant” to describe persons with asylum-seeker or similar backgrounds. The terms asylum-seeker and refugee have acquired stigma in the UK [37]. The PPI representatives also explained that once asylum has been granted, many individuals prefer to leave such labels behind; they want to shed the identity of refugee in order to start their new lives. For this reason, our recruitment materials were tailored to describe actions rather than address someone by identity, for example, asking potential participants “Have you ever sought asylum?” rather than ask them if they have refugee or asylum-seeker status. Table 3 lists some other aspects of how input by the PPI representatives influenced the proposed research plan.

**Table 3:** Additional examples of how aspects of research design were influenced by PPI input

Consideration	Design feature prompted by PPI input
Design of recruitment poster and materials	Culturally neutral without human representation
Data confidentiality and safety	Explicit descriptions of data protection and confidentiality measures in patient information sheet
Interviewer style, training	A personalized but professional manner was developed, to try to make the interviewee feel that their individual story was highly valued
Gender preference	Provision was made for interviewees to have a male or female interviewer, whichever they preferred
Consent form	Own copy to be supplied to participant (with thank you letter)
Location of interviews	Opportunity provided to conduct interviews at city centre location familiar to potential participants
Post interview materials	Thank you letter for participation

## Discussion

Although the background of the initial research team (and even of one of the PPI representatives) was overwhelmingly in quantitative science (mostly mathematical and epidemiological modelling), the most promising area of researched that emerged from our dynamic development process was highly qualitative, dependent on the complexities of human psychology and behavior. None of the investigators had a forced migrant or even ethnic minority background. It was essential to recruit PPI representatives who could provide more diversity of perspectives, particularly if we wanted to address sensitive health topics affecting forced migrants.

As part of our dynamic development process, a process which ideally includes small scale testing, the planned research is a relatively small study (just twelve interviews planned). A more ambitious study may follow. By incorporating the observations from this small study, an expanded set of interviews on the emotional resilience topic could be undertaken (in a larger city) with more people of forced migrant background. Alternatively or additionally, we may use the themes revealed by our interviewees as inputs to focus group discussions with staff of local organisations that work to support forced migrants. Author and PPI representative JO who works for the Norwich Red Cross, has indicated that discussions about the mismatch between what participants believe are their most successful strategies for adapting to life in the UK compared to what their support workers think are successful strategies, could provide fresh insights about support gaps and points where communication could be improved.

### Limitations

It is valid to say that our PPI representatives had key and unusually influential roles in setting specific research objectives, as well as study design, recruitment and preparation. Nevertheless, the objectives were somewhat restricted by the mandate agreed with the funding body (NIHR) that the research needed to provide evidence for reducing the public health impacts of complex events. There are other opportunities (via lobbying and political action) for patient or indeed forced migrant representative groups to influence public health policy with regard to setting health research agendas.

This work was undertaken before the publication of the GRIPP2 guidelines for reporting patient and public involvement in research [38] and may not conform to all aspects of best practice. We did not adopt a structured framework for PPI involvement. We did, however, loosely adopt the EPR HPRU’s published PPI/Engagement strategy and asked a selection of researchers and both public advisors to provide PPI feedback; all responses were constructive and positive. There was no formal process to assess how much team members valued the public participation. All of the researcher authors (SL, PRH, CCH and JSB) had limited pedagogical introduction to PPI through past professional experience. Author JSB had also previously consulted PPI advisors for an unrelated project. There was potential support from the EPR HPRU lead for PPI. We were able to refer to the NIHR’s web based INVOLVE resources. However, the researchers had no explicit practical hands-on training in PPI and the representatives had no previous similar experiences. It is possible that the research would have benefitted from more extensive training.

Our PPI representatives were not involved in analysis of the data collected by the research study that they helped to plan, although they had opportunity to advise on how to analyze the data. It has been argued that patient representatives reviewing real data can add richness to analysis of data [39]. That would not have been appropriate in our context. The forced migrant community in Norfolk is relatively small and there are confidentiality concerns: our PPI representatives might recognize identifying details among our interviewees. This potential risk could have made it impossible for us to get ethical approval to run the interviews. Moreover, we appreciated the PPI representative input as “critical friends” who provided a voice for those who had a stake in our research design and results. Thus, the PPI representatives collaborated but also maintained independence to complain if they disliked our results. One way to counter these problems could be to recruit additional PPI representatives from outside our region, who might be able to access raw (if in part redacted) interview transcripts to act as co-researchers. Possibly this is how we could have different types of public advisors with different roles as research partners or critical friends.

It can be argued that our application of Swarm for project development was limited; ideally Swarm means ongoing revisions in incremental steps as implementation proceeds. We did not have the freedom to plan for possible significant revisions during implementation of the resilience study. That a full Swarm approach was not easily compatible with the process of gaining ethical approval to run our study: once granted, we were obliged to undertake the study as described in the application. However, as much as was practical, we have tried to use agile development ideas in the development process and the experience of the small resilience study will be able to inform any follow up research, a development strategy that is indeed compatible with Swarm. What we also did, that does fit with Swarm, was involve the PPI advisors at an early stage in setting objectives and deciding on the main methods of the research. PPI has sometimes been “tokenistic” [40], often limited to a single consultation session or only commenting on a few aspects of implementation. In contrast, we tried to give PPI representatives ongoing and extensive roles in multiple stages of our research planning.

## Conclusions

Using a Swarm approach that itself arises from theory on best working practices within a complex environment, we embraced a dynamic research development process that also incorporated patient and public involvement. Our PPI strategy ensured flexible and medium-long term contact with our group, resulting in major contributions from the PPI representatives in both setting the research objectives and in many aspects of implementation.

Agile development (Swarm) is unusual in public health or medicine [12, 16] but can be well-suited to rapidly changing situations where uncertainty is high, such as how to address the long-term consequences of the recent and ongoing migration crisis in Europe. Dynamic setting of research objectives using input by PPI representatives is desirable when trying to reduce the impacts of complex events. Our report demonstrates how PPI representatives can be integrated into a dynamic development process to set objectives and design research to sensitively address a difficult topic (mental illness) within a hard-to-reach and potentially marginalized social group.

## Abbreviations

CS: Complexity science or complex systems
PPI: Patient and public involvement
